# Meso-tetra(4-sulfonatophenyl)porphyrin silver/Ag nanoparticles/graphene-phase C_3_N_4_ with a sandwich-like structure and double-faced active centers *via* two-step room-temperature photocatalytic synthesis for ractopamine detection†

**DOI:** 10.1039/d1na00130b

**Published:** 2021-05-18

**Authors:** Xuehua Weng, Huiling Ye, Wenqiang Xie, Meihui Ying, Haibo Pan, Min Du

**Affiliations:** National & Local Joint Biomedical Engineering Research Center on Photodynamics Technology Fuzhou Fujian 350108 P. R. China; College of Chemistry, Fuzhou University Qishan Campus Fuzhou Fujian 350108 China hbpan@fzu.edu.cn; Fujian Key Lab of Medical Instrument and Pharmaceutical Technology, Fuzhou University Fuzhou Fujian 350108 China

## Abstract

Photochemical synthesis under visible light irradiation is a novel approach in the field of green chemistry, and composites with abundant active centers for electrochemical detection are highly attractive. Herein, a meso-tetra(4-sulfonatophenyl)porphyrin silver/Ag nanoparticles/graphene phase C_3_N_4_ nanosheets (Ag_2_TPPS_4_/AgNPs/ng-C_3_N_4_) material with a sandwich-like structure was synthesized using a two-step photocatalytic reaction at room temperature (25 °C). In the first visible light irradiation step and in the presence of a hole capture agent, Ag^+^ ions were photocatalytically reduced onto the surface of ng-C_3_N_4_ that was used as a photocatalyst. Then, the protons (H^+^) in the core of H_2_TPPS_4_ were substituted *in situ* by photo-oxidized Ag^+^ during the second visible light irradiation step and in the presence of an electron capture agent. The electrochemical response of Ag_2_TPPS_4_ and ng-C_3_N_4_ to ractopamine (RAC) results in the unique double-faced active centers of Ag_2_TPPS_4_/AgNPs/ng-C_3_N_4_, and the cores (AgNPs) are beneficial as bridges for the connection between Ag_2_TPPS_4_ and ng-C_3_N_4_ and for high-efficiency electron transfer. Hence, as-synthesized Ag_2_TPPS_4_/AgNPs/ng-C_3_N_4_ exhibits high sensitivity (a low detection limit of 5.1 × 10^−8^ M, S/N = 3.0), a wide linear range (1 × 10^−7^ to 1.2 × 10^−5^ M), and long-term stability. Based on the experimental verification of the electrochemical dynamics and electrostatic attraction at the interface between the dual-active-center surface and RAC, the electrochemical mechanism has been clarified. Specifically, in the multi-cycle oxidation of RAC, the blue shift of specific UV-vis peaks also confirms the electrocatalytic oxidation of the two terminal hydroxyl groups of RAC. In brief, Ag_2_TPPS_4_/AgNPs/ng-C_3_N_4_ with a sandwich-like structure and double-faced active centers enhances the detection sensitivity and electrocatalytic efficiency towards RAC.

## Abstract 2

Ag_2_TPPS_4_/AgNPs/ng-C_3_N_4_ with a sandwich structure was synthesized *via* two-step photocatalytic synthesis, and the composite with double-faced active sites and a conductor (AgNPs) was applied to the electrochemical detection of ractopamine.

## Graphical abstract

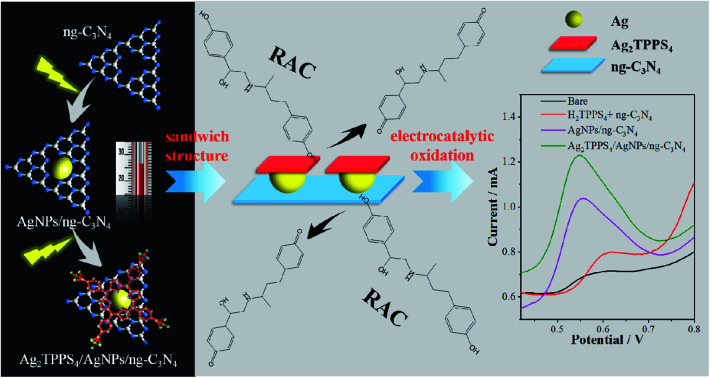

## Introduction

Both the increasing global demand for green synthesis and worldwide environmental concerns have triggered great attention towards searching for and focusing on novel ways to utilize alternative and renewable synthetic strategies, among which photocatalytic synthesis is known as one of the most abundant and easily accessible methods.^1^ Consequently, tremendous efforts have been made to utilize various metallic and organic catalysts capable of exhibiting photo-driven activity and mediating chemical reactions in the production of pharmaceuticals, fine chemicals and advanced materials.^2,3^ Thus, diverse composites have been prepared by this protocol, benefiting from the large amount of energy involved and the mild conditions.^4,5^ Kim and co-workers utilized UV illumination to excite carbon quantum dots with excellent electron-donating capability, which quickly induced the reduction of metal ions to the corresponding metal nanoparticles.^6^ Carbon-dot-supported silver (CD-Ag nanoparticles) was used to fabricate light-emitting diodes and polymer solar cells based on a solution-processable polymer. Following the early report of Zhou and co-workers, porphyrin-modified ultrathin Zr-MOF nanosheets were constructed to act as an effective photocatalyst for ^1^O_2_ generation and artemisinin production.^7^ In our previous report, a reduced graphene oxide (rGO)/PbTiO_3_ composite was successfully synthesized under UV irradiation and a highly sensitive electrochemisensor to detect pyrrole was fabricated.^8^ Note that photocatalytic synthesis accomplishes photocatalytic redox reactions by producing photogenerated electrons and holes through the use of a photocatalyst under mild conditions, which overcomes the problems of complicated chemical kinetics and saves energy *via* eliminating the need for high temperatures, which can also protect nanomaterials from damage.^9^

Porphyrins, a class of naturally occurring macrocyclic compounds such as hemoglobin and chlorophyll, are of significance in the metabolism of living organisms.^10^ The stable molecular structure of porphyrin is composed of four pyrrole rings linked *via* methine bridges, leading to aromatic character.^11^ Porphyrins are electron-rich organic molecules that are characterized by remarkably high extinction coefficients in the visible region.^12,13^ They are one of the most promising components of new materials for future electronic devices. For example, meso-tetra(4-sulfonatophenyl)porphyrin (H_2_TPPS_4_), a water-soluble porphyrin with hydrophilic groups and light-harvesting capacity,^14^ is beneficial for use in photocatalytic synthesis to prepare novel materials with specific features.

Graphene-phase C_3_N_4_ nanosheets (ng-C_3_N_4_) with a large surface area, which provides abundant reactive sites, and a short bulk diffusion length, which reduces the recombination probability of photo-excited charge carriers, are extremely advantageous for loading with porphyrin and to promote their catalytic performance.^15,16^ Nevertheless, the semiconductivity of ng-C_3_N_4_ hinders electron transfer between ng-C_3_N_4_ and H_2_TPPS_4_. Ag nanoparticles (AgNPs) have excellent electrocatalytic abilities and conductivity and are widely loaded onto the surfaces of supports in order to endow the resulting composites with new properties.^17,18^ The suitable conduction band (CB) and valence band (VB) (positioned at *ca.* −1.1 eV and *ca.* +1.6 eV *vs.* a normal hydrogen electrode (NHE), respectively^19,20^) of ng-C_3_N_4_ and the divalent vacancy in the center of the porphyrin ring, which is suitable for coordination with a metal, are able to exploit a photocatalytic synthesis strategy in which highly conductive AgNPs are anchored as a “bridge” to achieve connection and effective electron transportation between ng-C_3_N_4_ and H_2_TPPS_4_. The sandwich-like structure Ag_2_TPPS_4_/AgNPs/ng-C_3_N_4_ would be synthesized *via* a two-step photocatalytic process under visible light irradiation and at room temperature and be used as a unique double-faced electrocatalyst.

Ractopamine (RAC) is a synthetic beta-adrenergic agonist that can be used to treat congestive heart failure and muscular dystrophy, increase muscle mass, reduce small amounts of fat accumulation, decrease body weight, and enhance neonatal growth.^21^ Moreover, it can increase the daily weight gain of animals, improve feed utilization, and increase the protein content of animals. However, RAC has a stable structure and is not easily decomposed inside animals or humans. Large amounts of RAC residues in meat or dairy products will cause human poisoning, heart palpitations, muscle tremors and other negative effects. Pork containing RAC is now regulated by many countries.^22^ Subsequently, the monitoring and determination of RAC are extremely significant for food safety and human health. There are various highly selective and sensitive analytical methods for detecting RAC, such as high performance liquid chromatography, mass spectrometry, *etc.*,^23,24^ but their complicated operation, long time and high cost limit their wide application.^25,26^ Hence, it is necessary to develop a convenient, low-cost, and highly sensitive RAC detection method.

**Scheme 1:**
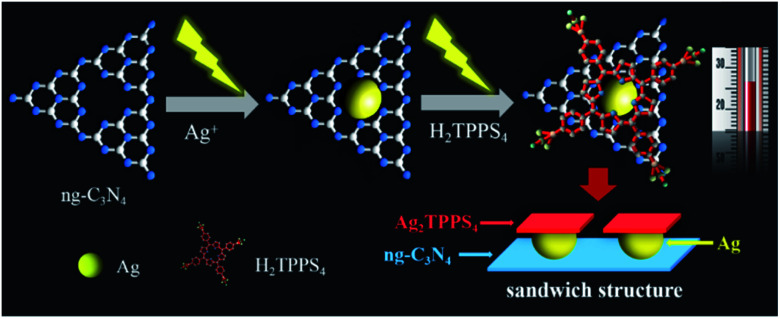
The photocatalytic synthesis processes of Ag_2_NPs/ng-C_3_N_4_ and Ag_2_TPPS_4_/AgNPs/ng-C_3_N_4_.

Here, a Ag_2_TPPS_4_/AgNPs/ng-C_3_N_4_ nanocomposite with a novel sandwich-like structure was fabricated *via* a facile and environmentally friendly two-step photocatalytic synthesis under visible light irradiation and at room temperature (Scheme 1). First, Ag^+^ ions are reduced by photogenerated electrons to form silver nanoparticles on the surface of ng-C_3_N_4_, which is used as a photocatalyst. Secondly, under the same irradiation, meso-tetra(4-sulfonatophenyl)porphyrin silver (Ag_2_TPPS_4_) was synthesized *via* an *in situ* center-substituted (ISCS) process, *i.e.*, the hydrogen atoms in the core of H_2_TPPS_4_ were replaced by the unsaturated silver ions on the surface of the AgNPs. Finally, the connection and effective electron transportation resulted in the sandwich structure and double-faced electrocatalytic activity of Ag_2_TPPS_4_/AgNPs/ng-C_3_N_4_. The new strategy is expected to simplify the preparation process, reducing the introduction of impurities and enhancing the electron transportation of the hybrid composite. Thereby, Ag_2_TPPS_4_/AgNPs/ng-C_3_N_4_ offers excellent conductivity, abundant electrocatalytic active centers, high specific surface area and a stable structure. In virtue of the above benefits, Ag_2_TPPS_4_/AgNPs/ng-C_3_N_4_ was applied as an electrochemical sensor for the efficient detection of RAC. The electrochemical analysis results demonstrated that the Ag_2_TPPS_4_/AgNPs/ng-C_3_N_4_ exhibited excellent sensitivity, a wide linear range and a low detection limit.

## Experimental

### Materials

Meso-tetraphenyl porphyrin (99.95%, 325 mesh) was obtained from URChem (Shanghai, China). RAC was purchased from Sigma-Aldrich (St. Louis, MO, USA). RAC stock solutions (1 mM) were prepared using ultra-pure water in all experiments. The working solutions were diluted from the stock solution using phosphate buffer solution (PBS, 0.1 M, pH 7.2). All other chemicals used were of analytical reagent grade, and were purchased from Beijing Chemical Reagent Company (Beijing, China) and used without further purification. Ultra-pure water was obtained from a Milli-Q plus water purification system (Millipore Co. Ltd., USA) (18 MΩ).

### Synthesis of ng-C_3_N_4_ nanosheets and meso-tetra(4-sulfonatophenyl)porphyrin (H_2_TPPS_4_)

Melamine was heated at 550 °C for 4 hours with a ramp rate of 5 °C min^−1^ to produce bulk g-C_3_N_4_. Then, 400 mg of ground bulk g-C_3_N_4_ was placed in an open ceramic container and was heated at 500 °C for 2 h with a ramp rate of 5 °C/min to obtain ng-C_3_N_4_.^41^

H_2_TPPS_4_ was synthesized using the Adleb method from a previous report.^27^ A mixed solution of 8.5 ml (83.4 mmol) of benzaldehyde and 190 ml of propionic acid was poured into a three-necked flask and refluxed at 136 °C. A mixed solution of 5.6 ml (80.7 mmol) pyrrole and 15 ml propionic acid was slowly added dropwise to the above mixture, which was maintained at a low boil for 30 min. After cooling the mixture to room temperature, 70 ml of anhydrous ethanol was added. The mixture was then filtered and washed with ethanol until the filtered solution was colorless, and the crude product was obtained. The crude product was dissolved in chloroform on a 5 cm silica gel column (200–300 mesh), and all the eluent was collected. 100 ml of methanol was added overnight. The bright purple crystals obtained by extraction were metal-free porphyrins (H_2_TPPS_4_).

The prepared metal-free porphyrin was sulfonated in chlorosulfonic acid. 2.7 ml of chlorosulfonic acid (98%) was placed in a 100 ml three-necked flask. 1 g of porphyrin powder was dissolved in 50 ml of the solvent 1,2-dichloroethane. The above solution was mixed in the three-necked flask and stirred at 120 °C for 4.5 h. After the stirring was complete and the temperature had spontaneously decreased to below 45 °C, the above solution was diluted with ice water, filtered and washed with distilled water to neutrality, and tested with AgNO_3_/HNO_3_ (dilute) solution until there was no HCl. The filter cake was dried in a vacuum at 70 °C for 48 h, and the resulting product was subjected to column separation. A basic alumina column was used, and the mobile phase was a mixture of water : methanol : acetone (7 : 2 : 1). The middle pink layer was collected, and the H_2_TPPS_4_ was obtained later use.

### Two-step synthesis of Ag_2_TPPS_4_/AgNPs/ng-C_3_N_4_ under visible light irradiation and at room temperature

A two-step photocatalytic process was applied to form the Ag_2_TPPS_4_/AgNPs/ng-C_3_N_4_ composite (Scheme 1). 30 mg of the as-synthesized g-C_3_N_4_ and 0.051 g AgNO_3_ were suspended in 20 ml of a mixture of water and isopropanol (3 : 1). Under nitrogen gas protection, the suspension was agitated and irradiated by visible light (300 W xenon arc lamp) for 60 min at room temperature (25 °C). Next, the suspension was centrifuged three times with water to remove Ag^+^ and dispersed again in a mixture of water and isopropanol (3 : 1). Note that isopropanol was used here as a photogenerated-hole capture agent. Subsequently, 20 ml of H_2_TPPS_4_ (0.22 mM) and *p*-benzoquinone (BQ, 0.92 mM, as an electron capture agent) were added to the newly prepared AgNPs/ng-C_3_N_4_ suspension and irradiated within 60 min under visible light in order to synthesize the Ag_2_TPPS_4_/AgNPs/ng-C_3_N_4_ suspension. It needs to be emphasized that these synthetic processes were carried out at room temperature (25 °C), and that the as-prepared Ag_2_TPPS_4_/AgNPs/ng-C_3_N_4_ was dried in a vacuum at 70 °C for 24 h without further sintering.

### Preparation of a RAC electrochemical sensor based on Ag_2_TPPS_4_/AgNPs/ng-C_3_N_4_

Prior to the preparation of the sensor, a glassy carbon electrode (GCE) was polished to mirror smoothness using a polishing cloth with 0.3 and 0.05 μm alumina powder, respectively. The GCE was then rinsed with deionized water, and ultrasonicated in deionized water and an ethanol bath, sequentially. 6 μl of Ag_2_TPPS_4_/AgNPs/ng-C_3_N_4_ was dropped onto the surface of the working GCE and dried at room temperature. Thus, the modified GCE with a uniform film coating (Ag_2_TPPS_4_/AgNPs/ng-C_3_N_4_/GCE) for detecting RAC was obtained.

### Characterization and electrochemical measurements

Fourier transform infrared spectroscopy (FT-IR) was conducted using a Nicolet Nexus 670 FT-IR spectrophotometer. ^1^HNMR was carried using a nuclear magnetic resonance spectrometer (NMR, Avance 400, Switzerland). The suspension in a capped quartz reactor was photo-irradiated with a xenon lamp (Model: PLS-SXE 300C, Perfect Light, China. Optical Filter, UVREF, *λ* = 200–400 nm). UV-vis measurements were performed using a UV-1800 SHIMADZU spectrophotometer. The fluorescence spectra were obtained at room temperature using a steady/transient fluorescence spectrophotometer (FLS 920, Edinburgh, U.K.). The morphology of the samples and their energy spectra were characterized using field-emission scanning electron microscopy (FE-SEM, Hitachi S4800, Japan). High-resolution transmission electron microscope (HRTEM) images were collected using a Tecnai G2 F20 S-TWIN, 200 kV (FEI Company, USA). Cyclic voltammetric (CV) and differential pulse voltammetry (DPV) plots were obtained using a CHI 660D electrochemical workstation (CH Instrument Company, Shanghai, China). A three electrode cell system was utilized with a modified GCE as the working electrode, Ag/AgCl as the reference electrode, and a platinum wire as the counter electrode. CV and DPV measurements of Ag_2_TPPS_4_/AgNPs/ng-C_3_N_4_ were carried out at room temperature (25 °C).

## Results and discussion

### Structure and morphology of Ag_2_TPPS_4_/AgNPs/ng-C_3_N_4_ throughout the two-step photocatalytic synthetic processes

**Fig. 1:**
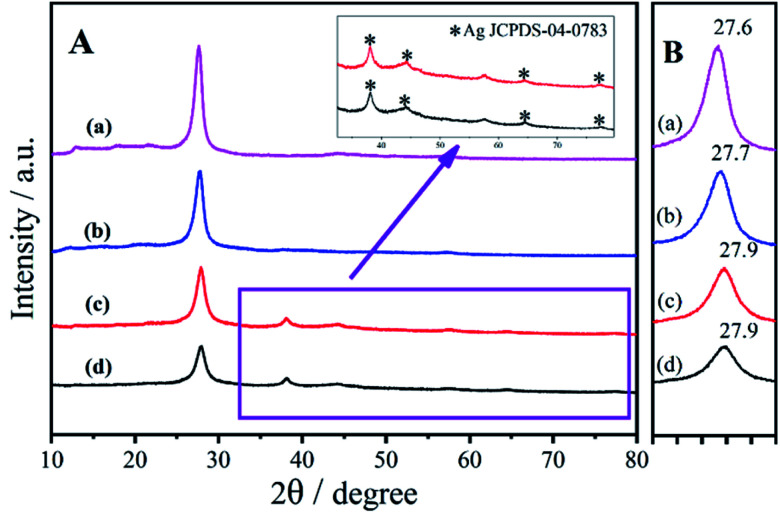
(A) XRD patterns of (a) bulk g-C_3_N_4_, (b) ng-C_3_N_4_, (c) AgNPs/ng-C_3_N_4_, and (d) Ag_2_TPPS_4_/AgNPs/ng-C_3_N_4_, and (B) the XRD patterns from 27–28°.

The structure of the ng-C_3_N_4_ used as the photocatalyst during the first photoreduction is important to the subsequent product in the two-step synthetic process. In order to observe the effect of thermal stripping, the morphology of g-C_3_N_4_ before and after calcination was analyzed using SEM (Fig. S1†). The bulk g-C_3_N_4_ before stripping exhibits accumulation and stacking, which is caused by the bulk g-C_3_N_4_ layers being closely bonded to each other (Fig. S1A†). After the process of thermal stripping (500 °C for 2 h), thin layers of g-C_3_N_4_ on both sides of bulk g-C_3_N_4_ are separated by heat under the high-temperature conditions (500 °C, 2 h), inducing thinner g-C_3_N_4_. Finally, the exfoliated g-C_3_N_4_ appears as a graphene-like flake, *i.e.*, ng-C_3_N_4_ (Fig. S1B†). Additionally, the lamellar structure of ng-C_3_N_4_ after the peeling process remains unchanged, as can be determined from their FT-IR spectra (Fig. S2†). Both the bulk g-C_3_N_4_ and ng-C_3_N_4_ samples display the typical vibration peaks of graphitic carbon nitride. The peak at 807 cm^−1^ is associated with the characteristic breathing mode of triazine units (Fig. S2A†).^28^ The broad band at approximately 3000 cm^−1^ corresponds to the stretching modes of terminal –NH_2_ or –NH groups (Fig. S2A†). The peaks at 1230, 1300 and 1390 cm^−1^ are ascribed to the typical stretching modes of aromatic C–N heterocycles, while the peaks at 1530 and 1625 cm^−1^ are assigned to the double bond C=N stretching vibration (Fig. S2B†). Meanwhile, the XRD pattern for g-C_3_N_4_ gives low-angle reflection peaks at 27.6° stemming from the g-C_3_N_4_ produced by stacking between slices (Fig. 1A(a)). The slight shift from 27.6° to 27.7° after calcination indicated that a number of layers of ng-C_3_N_4_ pieces stacked against each other (Fig. 1B).^29^ The NMR and UV-vis spectra of the as-prepared H_2_TPPS_4_ used as a precursor in the second photocatalytic process were measured (Fig. S3 and S4†). The ^1^HNMR spectrum (Fig. S3†) of H_2_TPPS_4_ in D_2_O exhibits two broad resonances at 7.08 and 8.69 ppm, which were ascribed to the β pyrrole protons. The two peaks at 7.54 and 8.13 ppm are attributed to the presence of *meta* and *ortho* phenyl protons, respectively.^30^ The behaviour of pure H_2_TPPS_4_ in a mixed liquid (water : isopropanol = 3 : 1) in the UV-vis spectrum (Fig. S4†) is identified according to its specific nature with a Soret band at 413 nm and Q-band absorption at 515, 552, 579 and 633 nm. Therefore, high-purity H_2_TPPS_4_ was definitely prepared.

For the systemic analysis for each product in the two-step photocatalytic process, XRD patterns were employed to characterize the nanostructure of AgNPs/ng-C_3_N_4_ and Ag_2_TPPS_4_/AgNPs/ng-C_3_N_4_, as shown in Fig. 1. In the first photocatalytic process in the presence of the hole capture agent (isopropanol), Ag^+^ was reduced to give AgNPs. In this process, Ag^+^ in AgNO_3_ was just reduced by the photogenerated electrons near the photocatalyst, ng-C_3_N_4_, so the AgNPs were loaded onto the surface of ng-C_3_N_4_. The XRD pattern of AgNPs/ng-C_3_N_4_ (Fig. 1A(c)) clearly displays peaks at 38.2°, 44.4°, 64.6° and 77.6°, which are indicative of the (111), (200), (220) and (311) planes of AgNPs (JCPDS-04-0783), respectively, indicating the formation of crystal AgNPs on the surface of ng-C_3_N_4_. After the AgNPs are loaded on the ng-C_3_N_4_, the peak of ng-C_3_N_4_ is shifted from 27.7° to 27.9°, revealing a decreased interlayer spacing and layer stacking of g-C_3_N_4_. After the second photocatalytic process, due to the rare substitution of the Ag atoms at the surface of the AgNPs, the pattern of the AgNPs is almost unchanged, while the diffracted intensity of ng-C_3_N_4_ is lower. In addition, the presence of the element Ag in the prepared nanocomposite is also confirmed by EDS (Fig. S5B†). After the second visible light irradiation, the patterns of ng-C_3_N_4_ and AgNPs remain unchanged due to the very low level of oxidation of the AgNPs to Ag_2_TTPS_4_.

**Fig. 2:**
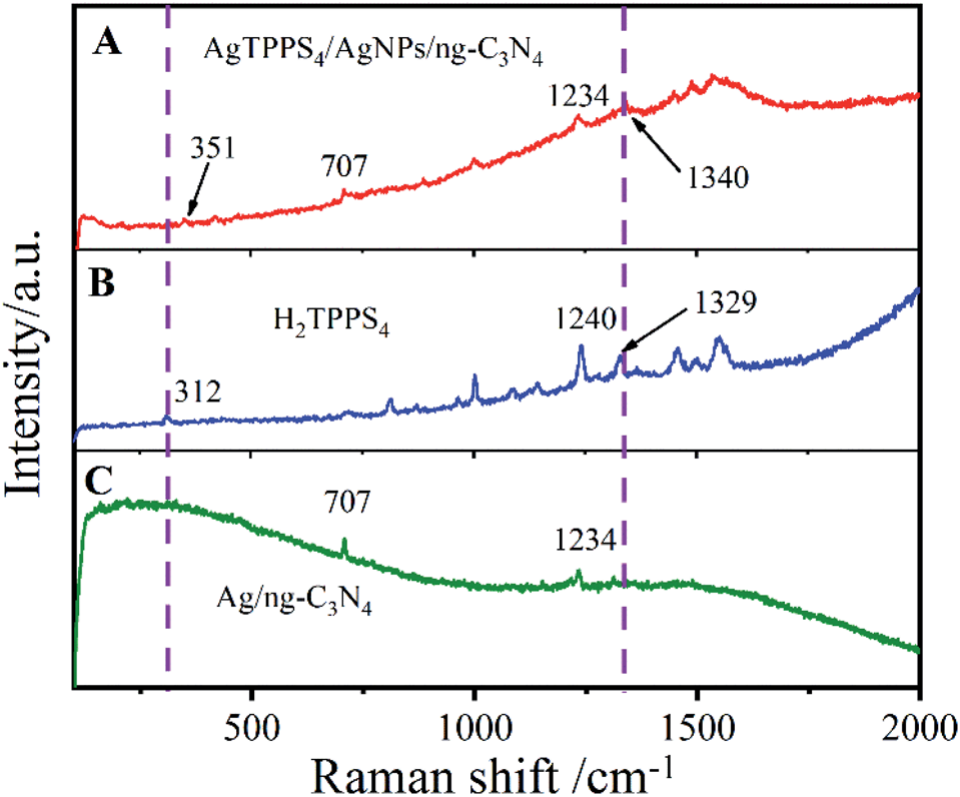
Raman spectra of (A) Ag_2_TPPS_4_/AgNPs/ng-C_3_N_4_, (B) H_2_TPPS_4_, and (C) Ag/ng-C_3_N_4_.

The Raman spectra of H_2_TPPS_4_, AgNPs/ng-C_3_N_4_ and Ag_2_TPPS_4_/AgNPs/ng-C_3_N_4_ were measured to confirm the interfacial characteristics of Ag_2_TPPS_4_/AgNPs/ng-C_3_N_4_ (Fig. 2). A weak Raman band in the low-frequency region is observed at 312 cm^−1^, corresponding to the C_α_C_m_C_α_ bending coordinate (Fig. 2A). The Raman band of H_2_TPPS_4_ at 1550 cm^−1^ is assigned to the vibration *ν*(C_α_/C_m_), while the bands at 1494 cm^−1^ and 1455 cm^−1^ are *ν*(C_α_/C_m_). The interactions between C–C bonds and porphyrin rings results in the 1566 cm^−1^ vibration peak. The Raman bands in 900–1400 cm^−1^ region are related to the stretching vibrations of C_α_C_β_/C_α_N bonds. The bands at 1534, 1450, and 1488 cm^−1^ (Fig. 2C) are ascribed to the *ν*(C_β_/C_β_) and *ν*(C_α_/C_m_) of H_2_TPPS_4_, which are slightly changed due to the coplanar conformation of porphyrins as depicted above.^31^ It is worth noting that the peak at 312 cm^−1^ caused by C_α_C_m_C_α_ bending is shifted to 351 cm^−1^ in the Ag_2_TPPS_4_/AgNPs/ng-C_3_N_4_ spectrum. Additionally, the peaks at 1240 and 1329 cm^−1^ disappear and a new peak at 1340 cm^−1^ appears, which further confirms that the introduction of Ag^+^ ions in a delocalized state changes the bending mode of the porphyrin rings.^32^ Characteristic bands at 707 and 1234 cm^−1^ clearly demonstrate the presence of AgNPs/ng-C_3_N_4_, indicating the successful connection of Ag_2_TPPS_4_ and AgNPs/ng-C_3_N_4_.

**Fig. 3:**
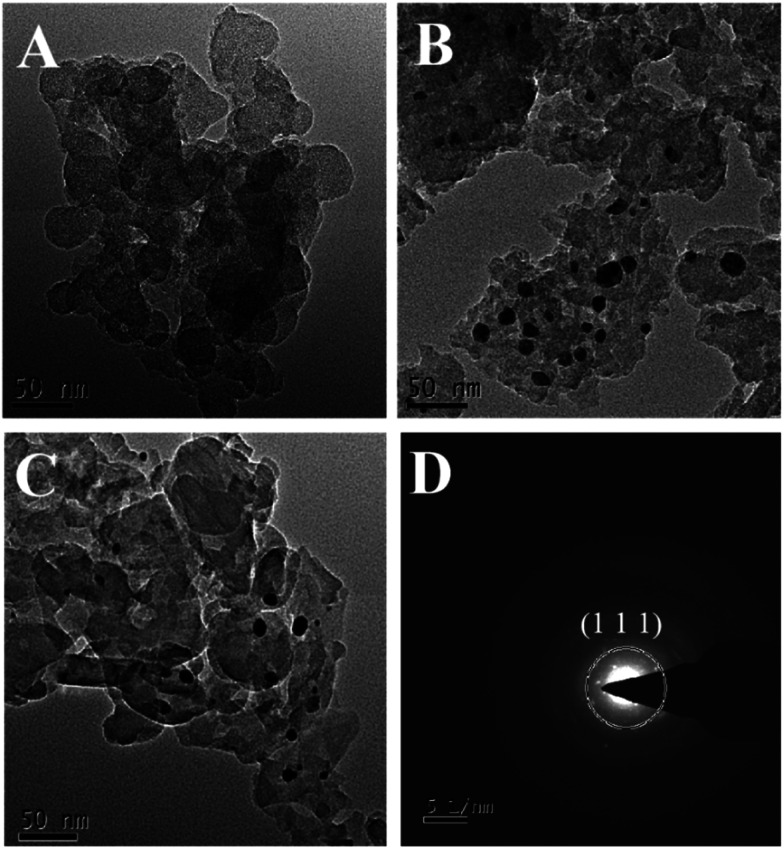
HRTEM images of (A) ng-C_3_N_4_, (B) AgNPs/ng-C_3_N_4_, and (C) Ag_2_TPPS_4_/AgNPs/ng-C_3_N_4_, and (D) SAED patterns of Ag_2_TPPS_4_/AgNPs/ng-C_3_N_4_.

The morphology and microstructure of ng-C_3_N_4_, AgNPs/ng-C_3_N_4_, Ag_2_TPPS_4_/AgNPs/ng-C_3_N_4_ were characterized with the HRTEM technique (Fig. 3). The results illustrate that ng-C_3_N_4_ appears in flakes (Fig. 3A). The AgNPs are inhomogeneously distributed with an average size of 20 nm on the ng-C_3_N_4_ (Fig. 3B). Then, H_2_TPPS_4_ was introduced into AgNPs/ng-C_3_N_4_. Under the second irradiation, Ag_2_TPPS_4_ was synthesized by means of an ISCS process, *i.e.*, the hydrogen atoms in the core of H_2_TPPS_4_ are substituted by the unsaturated silver ions on the surface of AgNPs, forming the Ag_2_TPPS_4_/AgNPs/ng-C_3_N_4_ composite (Fig. 3C). The morphology of the composite (Ag_2_TPPS_4_/AgNPs/ng-C_3_N_4_) has no significant change compared with that of AgNPs/ng-C_3_N_4_, and the size of the AgNPs of the former is similar to that of the latter. The reason for this is that the Ag_2_TPPS_4_ molecules are synthesized at the surface of AgNPs. Finally, a sandwich-like structure with AgNPs at the core of ng-C_3_N_4_ and Ag_2_TPPS_4_ (Scheme 1) was obtained. The two microcyclic electrochemical catalysts, ng-C_3_N_4_ and Ag_2_TPPS_4_, are beneficial to the high-efficient catalytic oxidation towards RAC, as shown later. In addition, the weak regular diffraction spots and diffraction rings with different radii in the SAED pattern (Fig. 3D) are assigned to the AgNP (111) crystal facets and the Ag_2_TPPS_4_/AgNPs/ng-C_3_N_4_ composition with a low crystallinity state. Furthermore, elemental mappings reveal that the elemental distributions of Ag are highly uniform (Fig. S9†), demonstrating the successful synthesis of Ag_2_TPPS_4_ by the ISCS process.

### Photophysicochemical characteristics of ng-C_3_N_4_, AgNPs/ng-C_3_N_4_ and Ag_2_TPPS_4_/AgNPs/ng-C_3_N_4_

**Fig. 4:**
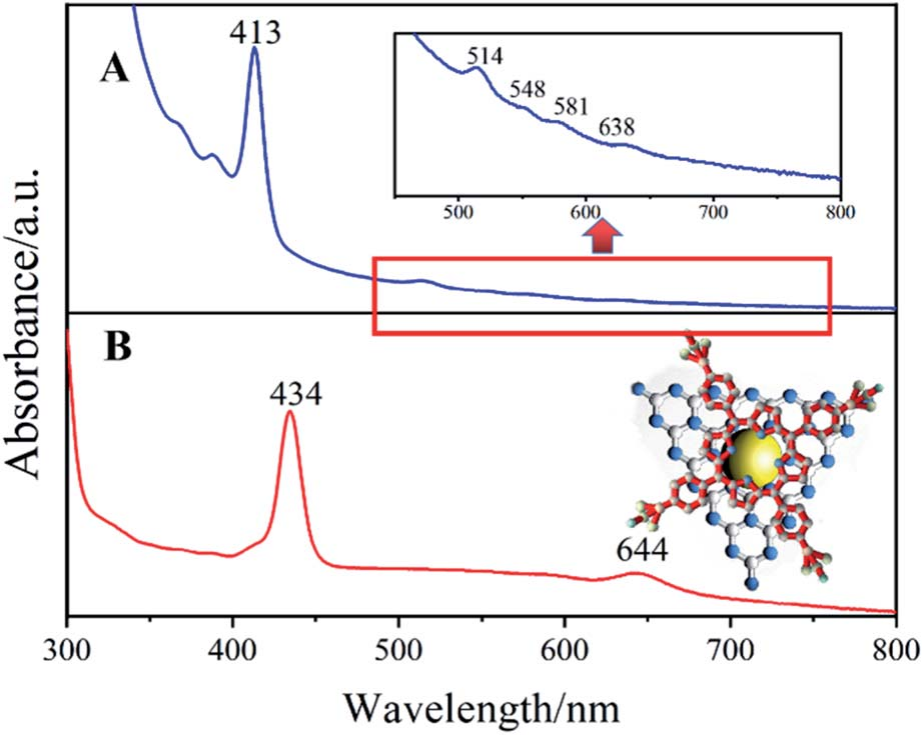
UV-vis spectra of Ag_2_TPPS_4_/AgNPs/ng-C_3_N_4_ in BQ solution (A) before and (B) after visible light irradiation. Inset: a structural diagram of Ag_2_TPPS_4_/AgNPs/ng-C_3_N_4_.

To verify the ISCS process of H_2_TPPS_4_ on the surface of AgNPs/ng-C_3_N_4_, a suspension of Ag_2_TPPS_4_/AgNPs/ng-C_3_N_4_ before and after visible light irradiation has been characterized using UV-vis spectra (Fig. 4). The initial Soret band for Ag_2_TPPS_4_/AgNPs/ng-C_3_N_4_ is recorded at 413 nm, while a new red-shifted Soret band at 434 nm is observed after irradiation, which is related to the molecular flattening of the porphyrin upon interaction with AgNPs/ng-C_3_N_4_ surfaces.^33^ Moreover, a new band for Ag_2_TPPS_4_/AgNPs/ng-C_3_N_4_ is observed at 644 nm, accompanied by the disappearance of four free base porphyrin bands at 514, 548, 584 and 638 nm, confirming that the center of H_2_TPPS_4_ is substitutively coordinated to Ag^+^ ions to assemble on the surface of AgNPs/ng-C_3_N_4_ and form Ag_2_TPPS_4_/AgNPs/ng-C_3_N_4_*via* the ISCS route.^34^ In the presence of benzoquinone as an electron capture agent, in the AgNPs/ng-C_3_N_4_, the photogenic holes partially oxidized the AgNPs (Ag^0^ + photoexcited holes → Ag^+^), inducing the replacement of H^+^ by Ag^+^. Thus, Ag_2_TPPS_4_ was synthesized *in situ* at the surface of the AgNPs, which caused the methyl pyridine group of the porphyrin to rotate (a two-dimensional plane conformation) to form a coplanar conformation.^35^ The deviation from the conformation extends the *p*-conjugation of the porphyrin molecule and enhances the resonance electron-withdrawing effect of the methylpyridinium groups.^36^ Hence, the gap between the highest occupied molecular orbital (HOMO) and the lowest unoccupied molecular orbital (LUMO) decreased, as illustrated by the consequent red shift (413 nm to 434 nm) in Fig. 4.

**Fig. 5:**
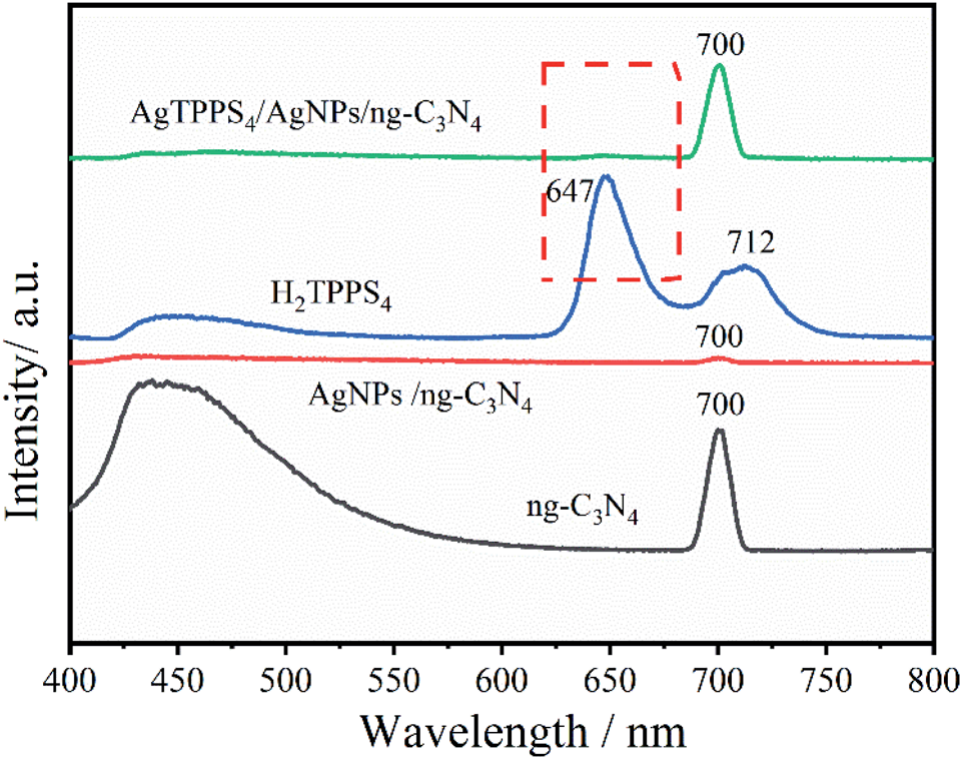
PL spectra of AgNPs/ng-C_3_N_4_, Ag_2_TPPS_4_/AgNPs/ng-C_3_N_4_, H_2_TPPS_4_, and ng-C_3_N_4_ in deionized water (the excitation light wavelength is 350 nm).

Fig. 5D shows the photoluminescence (PL) spectra of g-C_3_N_4_, H_2_TPPS_4_, Ag-C_3_N_4_ and Ag_2_TPPS_4_/AgNPs/ng-C_3_N_4_. Due to its strong photoluminescence, ng-C_3_N_4_ exhibits a high peak at 428 nm. The peak at 700 nm is second-order scattering (*λ*_SOS_), which is double its excitation wavelength (*λ*_ex_ = 350 nm). Both its photoluminescence and *λ*_SOS_ are greatly reduced after the first illumination. Nevertheless, the *λ*_SOS_ of Ag_2_TPPS_4_/AgNPs/ng-C_3_N_4_ increases after the second irradiation. From the theoretical calculation, the scattering intensity has been calculated to be,
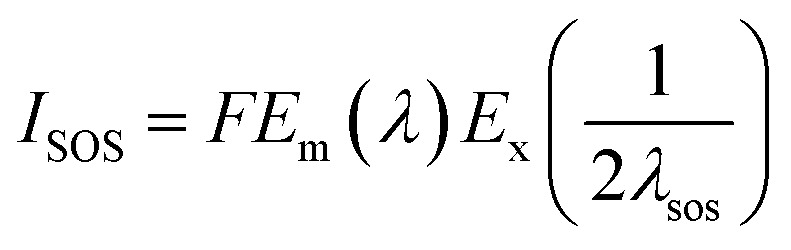
where *F* is characteristic of the materials, *E*_m_(*λ*) is the intensity distribution of the scattered light, and *E*_x_(*λ*) is the excitation spectrum of the scattered light.^37,38^ The alteration in the *λ*_SOS_ intensity indicates a change in the nature of the composite. Strong fluorescence quenching is detected for both ng-C_3_N_4_ and H_2_TPPS_4_ after anchoring AgNPs on ng-C_3_N_4_ and coupling H_2_TPPS_4_. The quenching after the first irradiation is caused by the photogenerated electron–hole pairs being effectively injected into the conduction band of the AgNPs through the LUMO of ng-C_3_N_4_. Moreover, the strong fluorescence quenching for H_2_TPPS_4_ after addition to AgNPs/ng-C_3_N_4_ results in the formation of a weak coordination complex, indicating the efficiency of electron transportation between Ag_2_TPPS_4_ and ng-C_3_N_4_ through the AgNPs. Thus, AgNPs here play a role as a conductor, accelerating the electrocatalytic process.

### Electrochemical characterization and electrochemical activity of the modified electrode

**Fig. 6:**
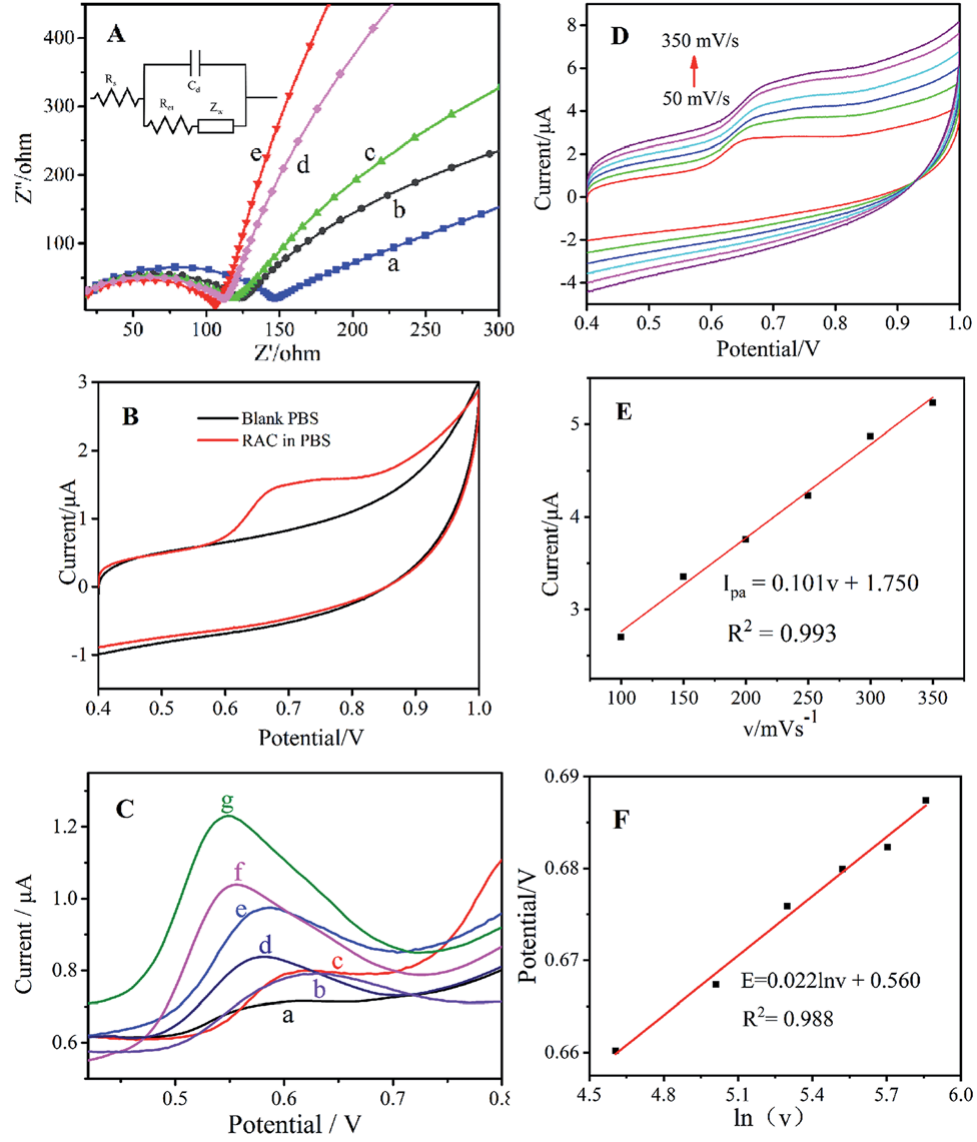
(A) EIS of (a) bare GCE, (b) ng-C_3_N_4_/GCE, (c) AgNPs/ng-C_3_N_4_/GCE, (d) Ag_2_TPPS_4_/GCE, and (e) Ag_2_TPPS_4_/AgNPs/ng-C_3_N_4_/GCE in 5.0 mM [Fe(CN)_6_]^3-/4-^; inset: the equivalent circuit. (B) CVs of a Ag_2_TPPS_4_/AgNPs/ng-C_3_N_4_-modified GCE in blank solution and in 0.1 M PBS (pH = 7) containing 1 × 10^−6^ M RAC. (C) DPV of (a) a bare GCE, (b) ng-C_3_N_4_/GCE, (c) H_2_TPPS_4_ + ng-C_3_N_4_/GCE, (d) AgNPs/GCE, (e) Ag_2_TPPS_4_/GCE, (f) AgNPs/ng-C_3_N_4_/GCE, and (g) Ag_2_TPPS_4_/AgNPs/ng-C_3_N_4_/GCE in PBS (0.1 M, pH 7.0) containing 1 × 10^−6^ M RAC. (D) CVs of Ag_2_TPPS_4_/AgNPs/ng-C_3_N_4_/GCE in PBS (pH 7.0) containing 1 × 10^−6^ M RAC at scan rates from 50 to 350 mV s^−1^. The effects of the scan rate on (E) the oxidation peak currents and (F) the potentials of RAC.

**Scheme 2:**
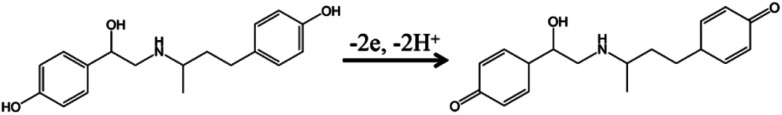
The electrochemical oxidation mechanism of RAC based on Ag_2_TPPS_4_/AgNPs/ng-C_3_N_4_/GCE.

Electrochemical impedance spectroscopy (EIS) can be utilized to analyse electrode process kinetics, electric double layers, material properties, *etc.* The Nyquist plot consists of a semicircle and a line with a 45° inclination for the electrode kinetics control process, and its radius can be used to describe the impedance of the electron transport process. The EIS of the different modified electrodes are shown in Fig. 6A, and an equivalent circuit diagram (*R*_s_: solution resistance, *C*_d_: capacitance, *R*_ct_: charge transfer resistance, and *Z*_w_: Warburg impedance^39–42^) is speculated in the inset. As shown in Fig. 6A, the bare GCE (Fig. 6A(a)) exhibits a high *R*_ct_ due to its poor electron transfer and weak electrochemical behaviour. The semicircle of ng-C_3_N_4_/GCE (Fig. 6A(b)) has large radius, demonstrating its high impedance based on its semiconductor characteristics. After being modified with AgNPs/ng-C_3_N_4_ (Fig. 6A(c)), the *R*_ct_ decreases slightly, which could be ascribed to the excellent conductivity of the AgNPs. When Ag_2_TPPS_4_ is modified on the GCE, the *R*_ct_ is also lower than for bare GCE, indicating that Ag_2_TPPS_4_ (Fig. 6A(d)) can be helpful to improve the conductivity of the sensor. The *R*_ct_ of the Ag_2_TPPS_4_/AgNPs/ng-C_3_N_4_/GCE (Fig. 6A(e)) is much smaller, suggesting the enhanced conductivity and electrochemical properties of Ag_2_TPPS_4_/AgNPs/ng-C_3_N_4_. It is supposed that the effect conjugation of the porphyrin structure further improves the efficiency of charge transfer. Cyclic voltammetry (CV) scans were recorded for Ag_2_TPPS_4_/AgNPs/ng-C_3_N_4_/GCE in blank PBS (pH = 7, 0.1 M) and PBS containing 1 × 10^−6^ M RAC (Fig. 6B). No obvious redox peak was observed for Ag_2_TPPS_4_/AgNPs/ng-C_3_N_4_/GCE in the blank PBS. However, a sensitive oxidation peak appeared at a potential of 0.68 V in PBS containing 1 × 10^−6^ M RAC, which was caused by the oxidation of RAC in PBS. It can be seen from the figure that there is no obvious reduction peak, indicating that the electrocatalytic reaction of RAC on the modified electrode is irreversible. To further investigate the electrocatalytic ability of the as-prepared nanocomposite toward RAC, DPV was employed to provide further detail, because it is an advantageous technique for obtaining higher sensitivity by eliminating the non-Faraday currents that occur in CV.^43^ Compared to the other electrodes in PBS (0.1 M, pH 7.0) containing 1 × 10^−6^ M RAC, the response signal of bare GCE to RAC is very faint throughout the scanning interval (Fig. 6C(a)). The ng-C_3_N_4_/GCE (Fig. 6C(b)) and the mixed H_2_TPPS_4_+ng-C_3_N_4_/GCE electrode (Fig. 6C(c)) have a weak response but electrocatalytically oxidize RAC. When AgNPs are modified onto the electrode, the peak current increases and the peak potential decreases (Fig. 6C(d)). In addition, Ag_2_TPPS_4_/GCE (Fig. 6C(e)) also has a catalytic effect toward RAC, and the peak current is higher than those of curves a–d. For AgNPs/ng-C_3_N_4_/GCE, the peak current (1.04 μA) increases significantly, indicating that AgNPs/ng-C_3_N_4_ has a better catalytic oxidation effect towards RAC. Note that when Ag_2_TPPS_4_/AgNPs/ng-C_3_N_4_ is modified onto the electrode, the clear increase in the peak current (1.23 μA) and the decrease of peak potential (0.55 V) indicate the reduction of the electrocatalytic activation energy, meaning that the special ring structure of Ag_2_TPPS_4_ effectively improves the electron transfer ability and overcomes the electrocatalytic barrier. The results reveal that both H_2_TPPS_4_ + ng-C_3_N_4_/GCE and AgNPs/ng-C_3_N_4_/GCE have an electrochemical response to RAC, and that the response of Ag_2_TPPS_4_/AgNPs/ng-C_3_N_4_ to RAC is significantly higher. Thereout, it is concluded that Ag_2_TPPS_4_/AgNPs/ng-C_3_N_4_ exerts double-faced active centers. Moreover, ng-C_3_N_4_/GCE, AgNPs/GCE and Ag_2_TPPS_4_/GCE all have electrocatalytic oxidation effects towards RAC, illustrating that the synergistic effect of the three nanomaterials enhances the electrocatalytic activity and response sensitivity. As a result, Ag_2_TPPS_4_/AgNPs/ng-C_3_N_4_/GCE possesses excellent electrocatalytic ability and extremely high response sensitivity. Additionally, the high conductivity of the AgNPs in the sandwich-like structure benefits the charge transfer between Ag_2_TPPS_4_ and ng-C_3_N_4_, thereby promoting the quick electrochemical response of the electrode to RAC. This is consistent with the fluorescence response characteristics. The potentials and peak current (*I*_pa_) responses of the Ag_2_TPPS_4_/AgNPs/ng-C_3_N_4_/GCE electrode at different scan rates from 50 to 350 mV s^−1^ are shown in Fig. 6D. Similarly, with increasing scanning speed (*v*), the oxidation peak potential shifted positively and *I*_pa_ improved. A linear relationship between the scan rate and *I*_pa_ can be observed in Fig. 6E. The fitted curve is *I*_pa_ = 0.011*v* + 1.60 (*R*^2^ = 0.993), which demonstrates that the electrode reaction of RAC on the Ag_2_TPPS_4_/AgNPs/ng-C_3_N_4_/GCE electrode is controlled by an adsorption process. Simultaneously, the peak potentials and scan rate have a linear relationship with a correlation (*R*^2^) of 0.993 (Fig. 6F). According to the Laviron equation (*E*_p_ = *E*_0_ + *RT* ln(*RTk*^0^/*αnF*)/*αnF* − *RT* ln *v*/*αnF*),^44^ the slope of the regression equation is *RT*/*αnF*, corresponding to the fact that two-electron transfer is involved in this process (Scheme 2).

To reveal the interfacial features and electrocatalytic mechanism for Ag_2_TPPS_4_/AgNPs/ng-C_3_N_4_ and RAC, zeta potential testing was carried out for five samples, as listed in Table S1.† The zeta potential of AgNPs/ng-C_3_N_4_ is −2.13 mV, indicating that the surface is negatively charged. When AgNPs/ng-C_3_N_4_ was successfully assembled with Ag_2_TPPS_4_ to form a sandwich-like structure, the zeta potential of Ag_2_TPPS_4_/AgNPs/ng-C_3_N_4_ was significantly more negative (−42.2 mV), indicating that the Ag_2_TPPS_4_/AgNPs/ng-C_3_N_4_ has more negative charges on its surface and great potential for electrostatic adsorption towards RAC, which is positively charged (1.26 mV). In addition, the zeta potentials of both AgNPs/ng-C_3_N_4_ + RAC (7.69 mV) and Ag_2_TPPS_4_/AgNPs/ng-C_3_N_4_ + RAC (−15.6 mV) are more positive, confirming that Ag_2_TPPS_4_/AgNPs/ng-C_3_N_4_ has double-sided electrostatic interaction. Therefore, the electrostatic attraction benefits the subsequent electrocatalytic process. The results suggest that the adsorption of the sandwich composite towards RAC through electrostatic interactions is conducive to the realization of high-sensitivity detection of RAC. To further investigate the electrocatalytic oxidation mechanism of RAC using Ag_2_TPPS_4_/AgNPs/ng-C_3_N_4_/GCE, differential pulse voltammetry (DPV) was applied in this work (Fig. S6†). The peak current gradually decreases after cyclic oxidation, confirming the decreased sensitivity toward RAC and the oxidation of RAC. Simultaneously, blue shifts at 223 nm and 280 nm in the UV-vis spectrum are displayed (Fig. S6B†) due to the oxidation of two terminal –OH groups of RAC to weaken the conjugated structure. Furthermore, a new low-intensity absorption band at 313 nm emerges after 250 rounds of cyclic oxidation, which corresponds to the n–π* electronic excitation of an α,β-unsaturated ketone.^45^ The above results demonstrate the consumption and conversion of RAC during the electrocatalytic detection process. Thus, they also prove the existence of double catalytic faces for a sandwich-like structure. Ag_2_TPPS_4_/AgNPs/ng-C_3_N_4_ is able to efficiently oxidize two hydroxyl groups and enhance the catalytic rate.

### Optimization of the conditions for the modified electrode and the electrochemical detection of RAC

In order to achieve a highly sensitive electrochemical sensor for RAC, the experimental conditions must be optimized. The accumulation potential of the peak current was measured from 0 to 0.4 V using a specific accumulation time (AT = 120 s) (Fig. S7A†), and the best accumulation potential was 0.2 V (Fig. S7B†). The effect of pH on the peak current of the Ag_2_TPPS_4_/AgNPs/ng-C_3_N_4_-modified electrode was studied over the range of pH 4 to 9 in PBS solution in the presence of RAC (1 × 10^−6^ M) with a potential of +0.2 V (Fig. S8A†). Fig. S8B† depicts a maximum current response at pH = 7. Therefore, for the subsequent experiments, PBS solution at pH 7 was used.

**Fig. 7:**
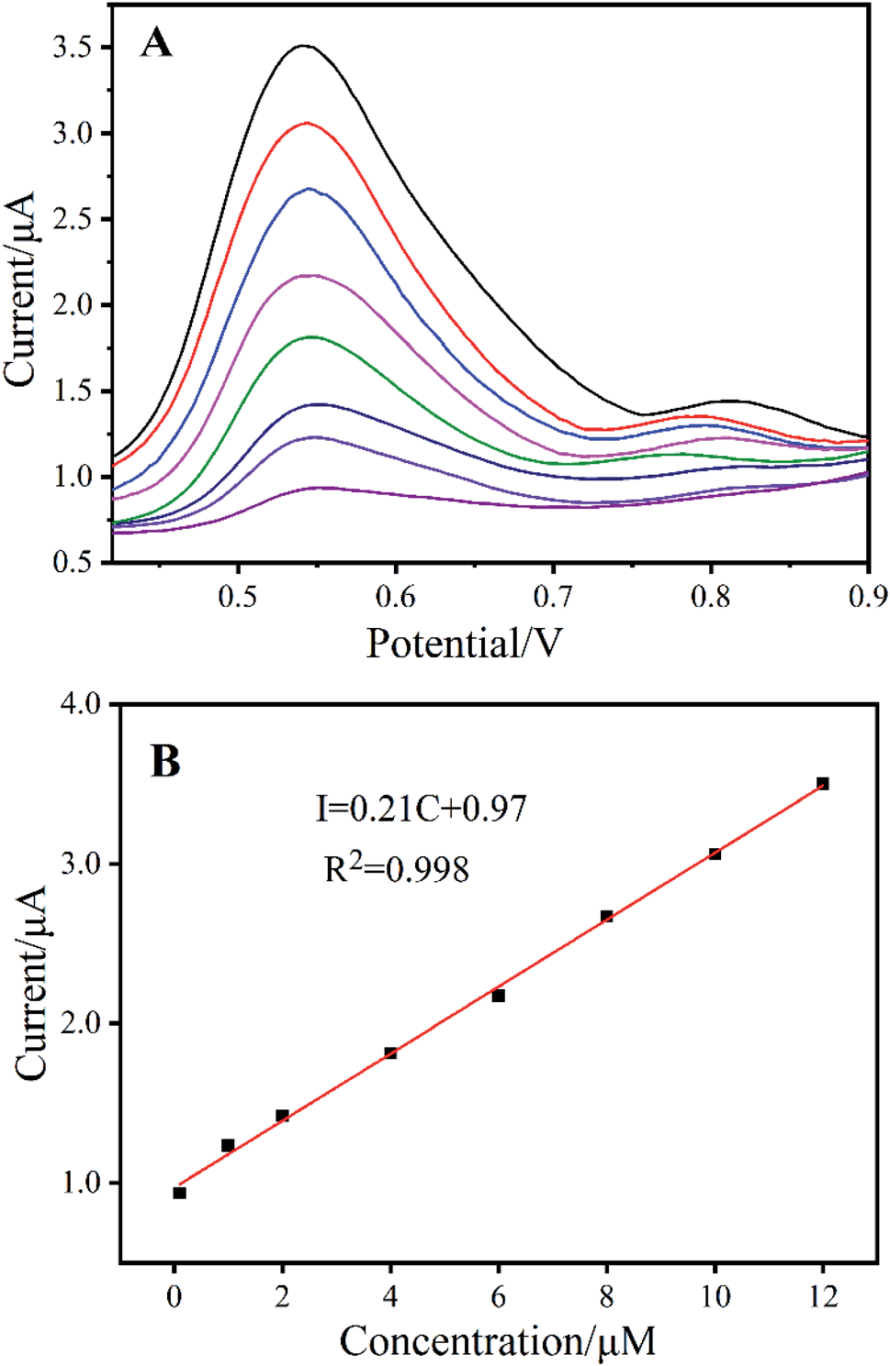
(A) DPV plots for Ag_2_TPPS_4_/AgNPs/mg-C_3_N_4_/GCE with different amounts of RAC in PBS (0.1 M, pH 7.0) (black: highest concentration; dark purple: lowest concentration). (B) The calibration curve showing the relationship between the RAC concentration and peak current.

The DPV curves in Fig. 7A show increasing oxidation peak current with increasing RAC concentration at approximately 0.54 V. The calibration data were obtained for RAC solutions under the optimum experimental conditions described in Fig. 7B, and the plot demonstrates linear behaviour in the range of 1 × 10^−7^ to 1.2 × 10^−5^ mol L^−1^ (*R*^2^ = 0.998) with a limit of 5.1 × 10^−8^ mol L^−1^ (S/N = 3). The linear range and the limit of detection of Ag_2_TPPS_4_/AgNPs/ng-C_3_N_4_/GCE were compared to those reported in the papers listed in Table S2,† which revealed that the Ag_2_TPPS_4_/AgNPs/ng-C_3_N_4_/GCE electrode has excellent sensitivity. Moreover, the detection sensitivity of the Ag_2_TPPS_4_/AgNPs/ng-C_3_N_4_/GCE electrode is superior to the CAC (Codex Alimentarius Commission, founded by FAO and WHO) standard (10 ppb).

### Selectivity, reproducibility, and stability of the modified electrode

The selectivity of the Ag_2_TPPS_4_/AgNPs/ng-C_3_N_4_/GCE was analyzed using different interferents, namely, glucose (Glu), dopamine (DA), ascorbic acid (AA), galactose (Gal), vanillin (Van), fructose (Fru), and acetaminophen (Ace). The relative current response of Ag_2_TPPS_4_/AgNPs/ng-C_3_N_4_/GCE in 50 μM RAC with the addition of 500 μM of the other chemicals is given in Fig. S10†. Ag_2_TPPS_4_/AgNPs/ng-C_3_N_4_/GCE shows no remarkable response towards the other interferents, indicating its outstanding selectivity for Ag_2_TPPS_4_/AgNPs/ng-C_3_N_4_/GCE. The reproducibility of the Ag_2_TPPS_4_/AgNPs/ng-C_3_N_4_/GCE was investigated by 20 cycles of DPV in PBS (pH = 7, 0.1 M) containing 1 × 10^−6^ M RAC. The peak current of oxidation did not decrease remarkably, and the relative standard deviation (RSD) was 3.56%, demonstrating the feasibility of the sensor. The stability of this alternative developed method was also investigated by measuring the current response at a fixed RAC concentration over a long period. The RSD of the stability of oxidation remains 3.7% after storage in air for 7 days, indicating that Ag_2_TPPS_4_/AgNPs/ng-C_3_N_4_/GCE possesses excellent stability. To examine the actual detection effect of the electrochemical sensor, the Ag_2_TPPS_4_/AgNPs/ng-C_3_N_4_/GCE was used to analyse three milk samples purchased from the local market. Recovery experiments were performed using the standard addition method. The value of recovery in milk is in the range from 92 to 114% (Table S3†), revealing that this new method is accurate and feasible.

## Conclusions

An environmentally friendly photochemical synthesis method was performed to prepare Ag_2_TPPS_4_/AgNPs/ng-C_3_N_4_ with a sandwich-like structure and double-faced active centres. In the first visible light irradiation step and in the presence of a hole capture agent, AgNPs were successfully anchored on the surface of ng-C_3_N_4_. Ag_2_TPPS_4_ was formed *via* an *in situ* centre-substituted (ISCS) process during the second irradiation step and with the addition of an electron capture agent. The core AgNPs were used as a “conductive bridge” connecting both ng-C_3_N_4_ and Ag_2_TPPS_4_ to achieve the formation of a sandwich-like and highly efficient double-faced electrocatalyst. This Ag_2_TPPS_4_/AgNPs/ng-C_3_N_4_ with a sandwich-like structure was utilized to construct a double-faced electrocatalytic platform for the nonenzymatic detection of RAC. Ag_2_TPPS_4_ and ng-C_3_N_4_ play the role of electrocatalysts, reducing the catalytic barrier and oxidizing the –OH groups of RAC. Based on the experimental results, the electrochemical sensor not only exhibits excellent electrocatalytic activity towards RAC with a broad linear range and low detection limit, but it also provides remarkable long-term stability. This can be ascribed to the high efficiency of charge transport, the double-sided electrocatalytic activity, the high specific surface area, and the synergistic effect of the hybrid composite. The successful application of this electrode demonstrates that modified nanocomposites provide a new platform for designing sensors to determine RAC sensitively.

## Author contributions

Xuehua Weng: writing – original draft, formal analysis. Huiling Ye: data curation. Wenqiang Xie: conceptualization, writing – original draft. Meihui Ying: formal analysis. Haibo Pan: supervision, conceptualization, methodology, writing – review & editing. Min Du: project administration.

## Conflicts of interest

There are no conflicts to declare.

## Supplementary Material

NA-003-D1NA00130B-s001
