# Supplementary material for: Meso-tetra(4-sulfonatophenyl)porphyrin silver/Ag nanoparticles/graphene-phase C3N4 with a sandwich-like structure and double-faced active centers via two-step room-temperature photocatalytic synthesis for ractopamine detection
Source: Nanoscale Adv. 2021 May 18;3(13):3900–8. doi: 10.1039/d1na00130b (PMC9419538; doi:10.1039/d1na00130b)
Supplement: NA-003-D1NA00130B-s001 [file NA-003-D1NA00130B-s001.pdf]

Electronic Supplementary Material (ESI) for Nanoscale Advances.
This journal is © The Royal Society of Chemistry 2021

Meso-tetra (4-sulfonatophenyl) porphyrin silver / Ag nanoparticles / graphene phase C_3N_4 with a sandwich-like structure and double-faced active centers via two-step photocatalytic room-temperature synthesis for ractopamine detection

Xuehua Weng [a,b], Huiling Ye [a,b], Wenqiang Xie[a,b], Meihui Ying[a,b], Haibo Pan[a,b,c]*, Min Du [c]

[a] *National & Local Joint Biomeidical Engineering Research Center on Photodynamics Technology, Fuzhou, Fujian 350108, P. R. China*

[b] *College of Chemistry, Qishan Campus, Fuzhou University, Fuzhou, Fujian 350108,, China*

[c] *Fujian Key Lab of Medical Instrument and Pharmaceutical Technology, Fuzhou University, Fuzhou, Fujian 350108, China*

*Corresponding authors

Telephone: 86-591- 22866127; Fax: 86-591-22866127

E-mail: hbpan@fzu.edu.cn (H. Pan)

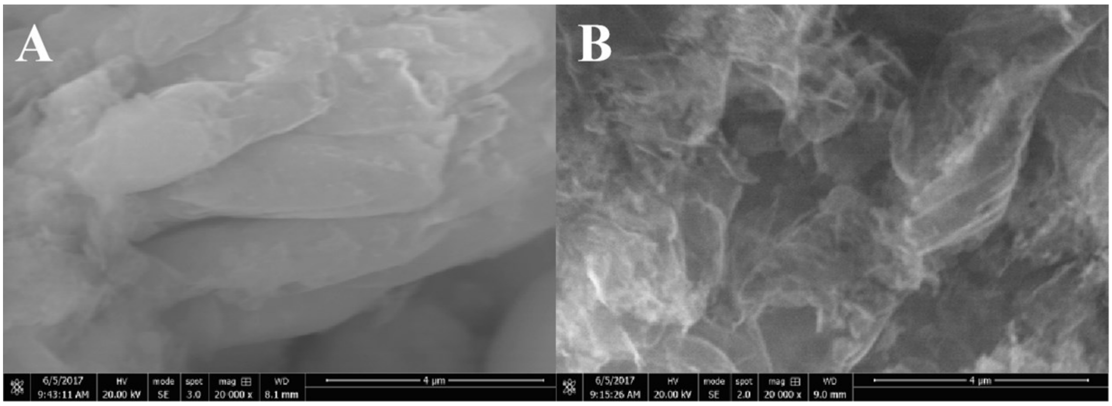

Fig. S1 SEM images of (A) bulk g-C_3N_4 and (B) ng-C_3N_4.

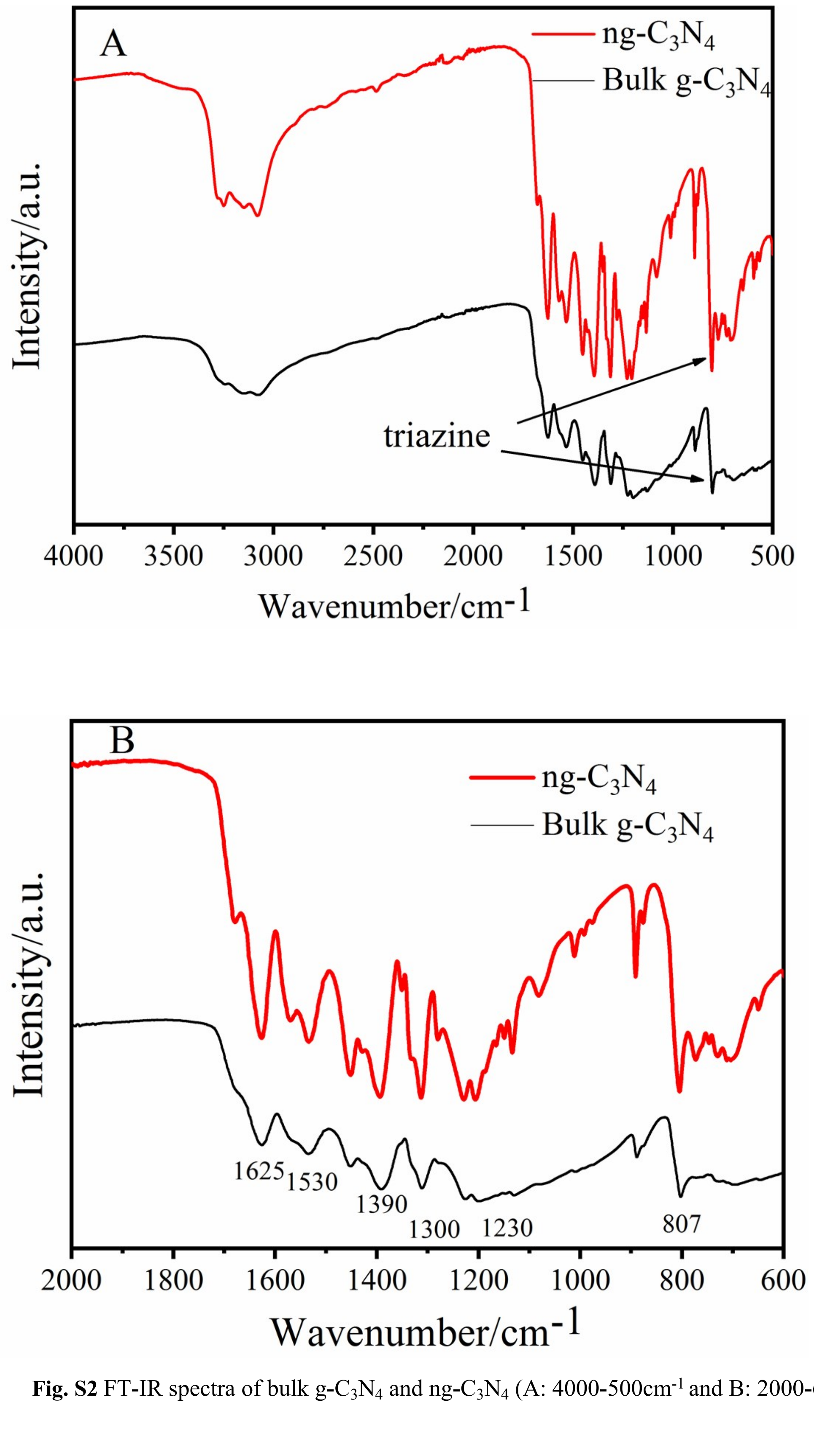

Fig. S2 FT-IR spectra of bulk g-C_3N_4 and ng-C_3N_4 (A: 4000-500cm^{-1} and B: 2000-600cm^{-1}).

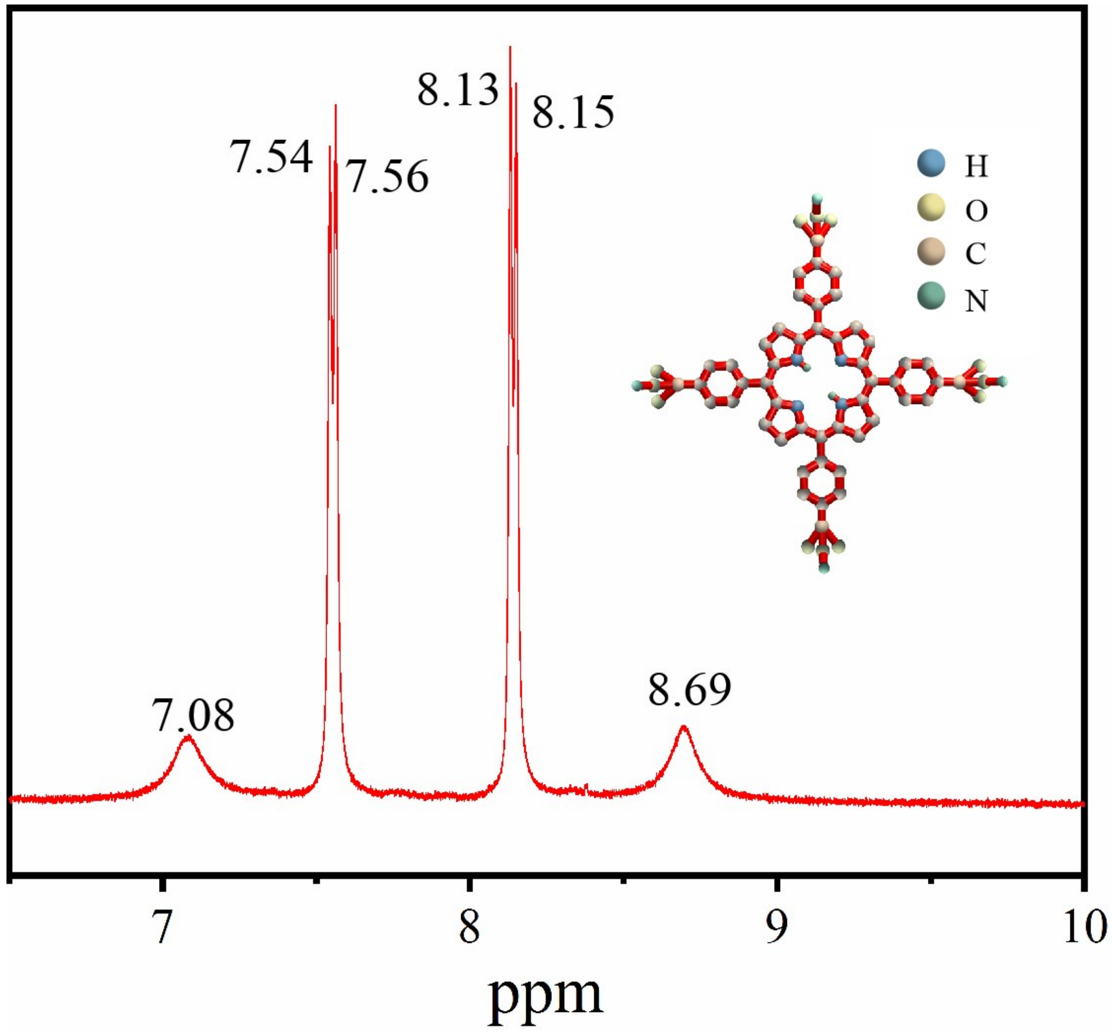

Fig. S3 ^{1}HNMR spectrum of H_2TPPS_4 in D_2O, insert: molecular diagram of H_2TPPS_4.

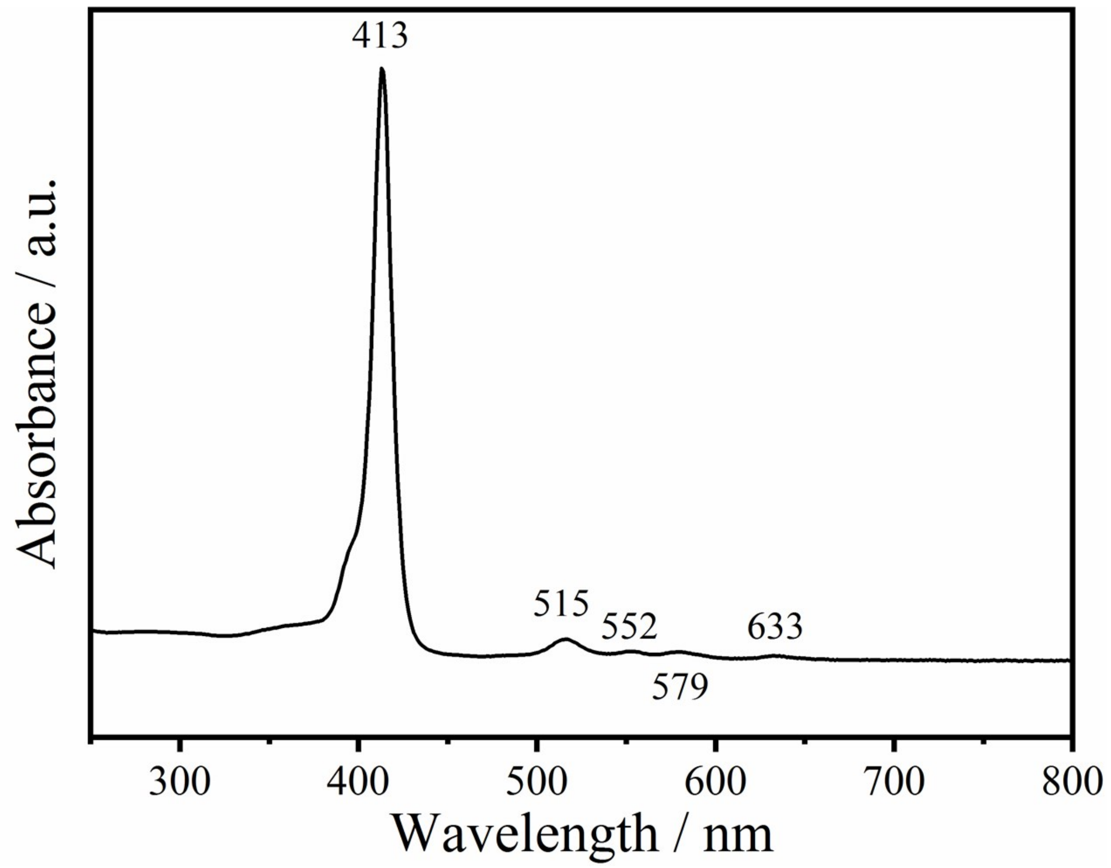

Fig. S4 UV-vis spectrum of H_2TPPS_4 in H_2O.

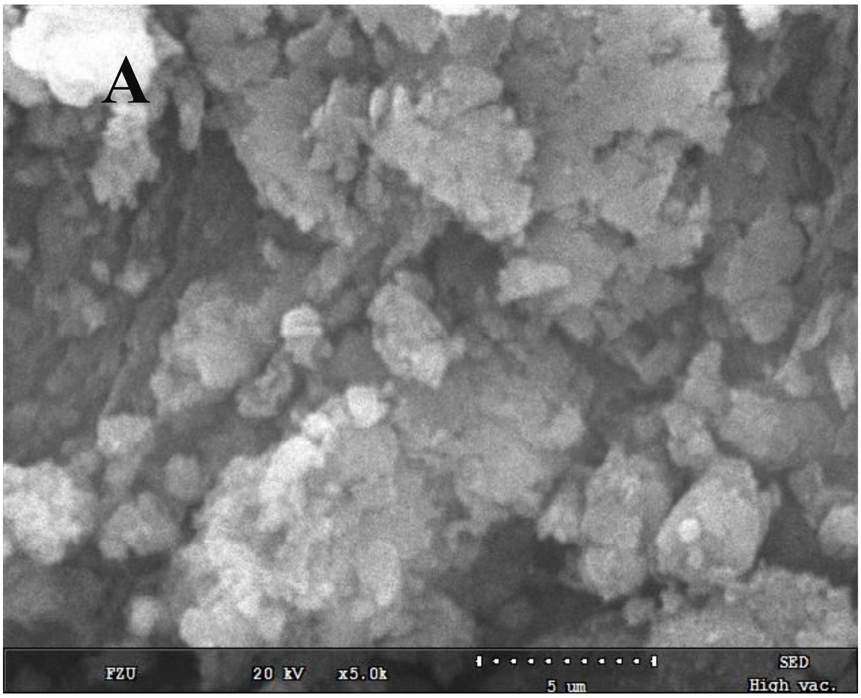

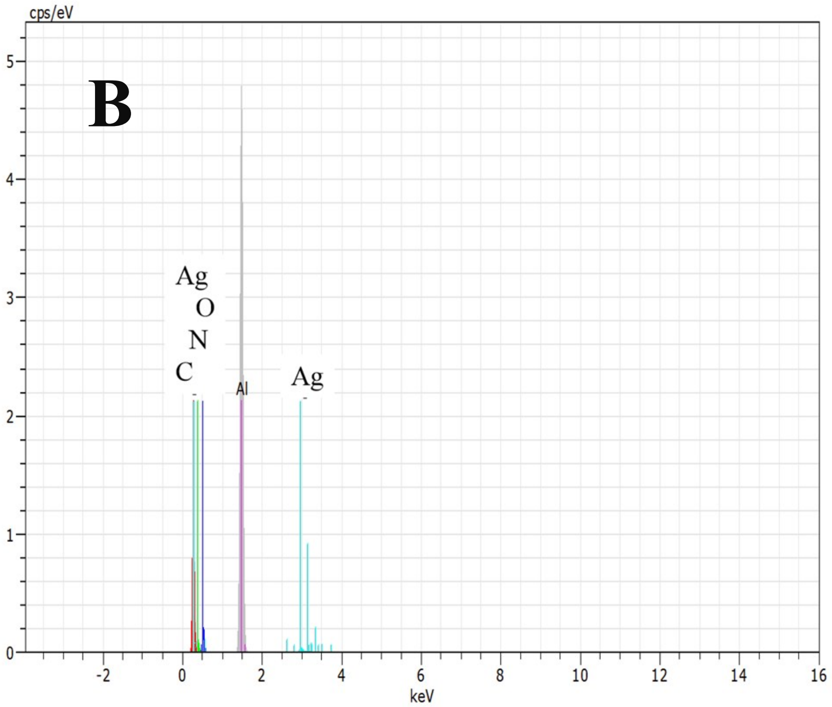

Fig. S5 (A) SEM images and (B) EDS of Ag/ng-C_3N_4.

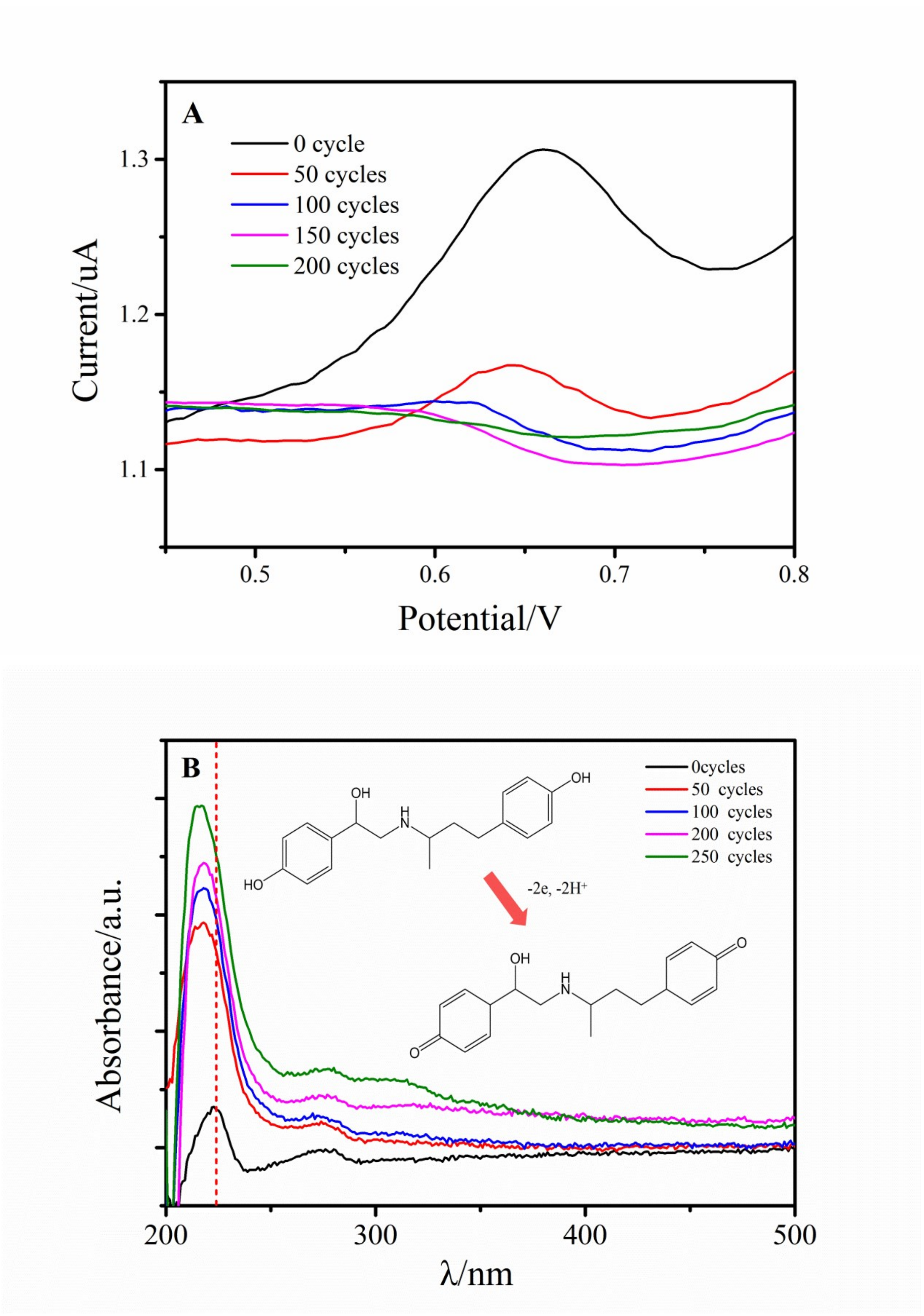

Fig. S6 (A) DPV curves of Ag_2TPPS_4/AgNPs/ng-C_3N_4/GCE in pH 7.0 PBS with 1×10^{-6} M RAC.

(B) UV-vis spectra for RAC after oxidized cycles in pH 7.0 PBS.

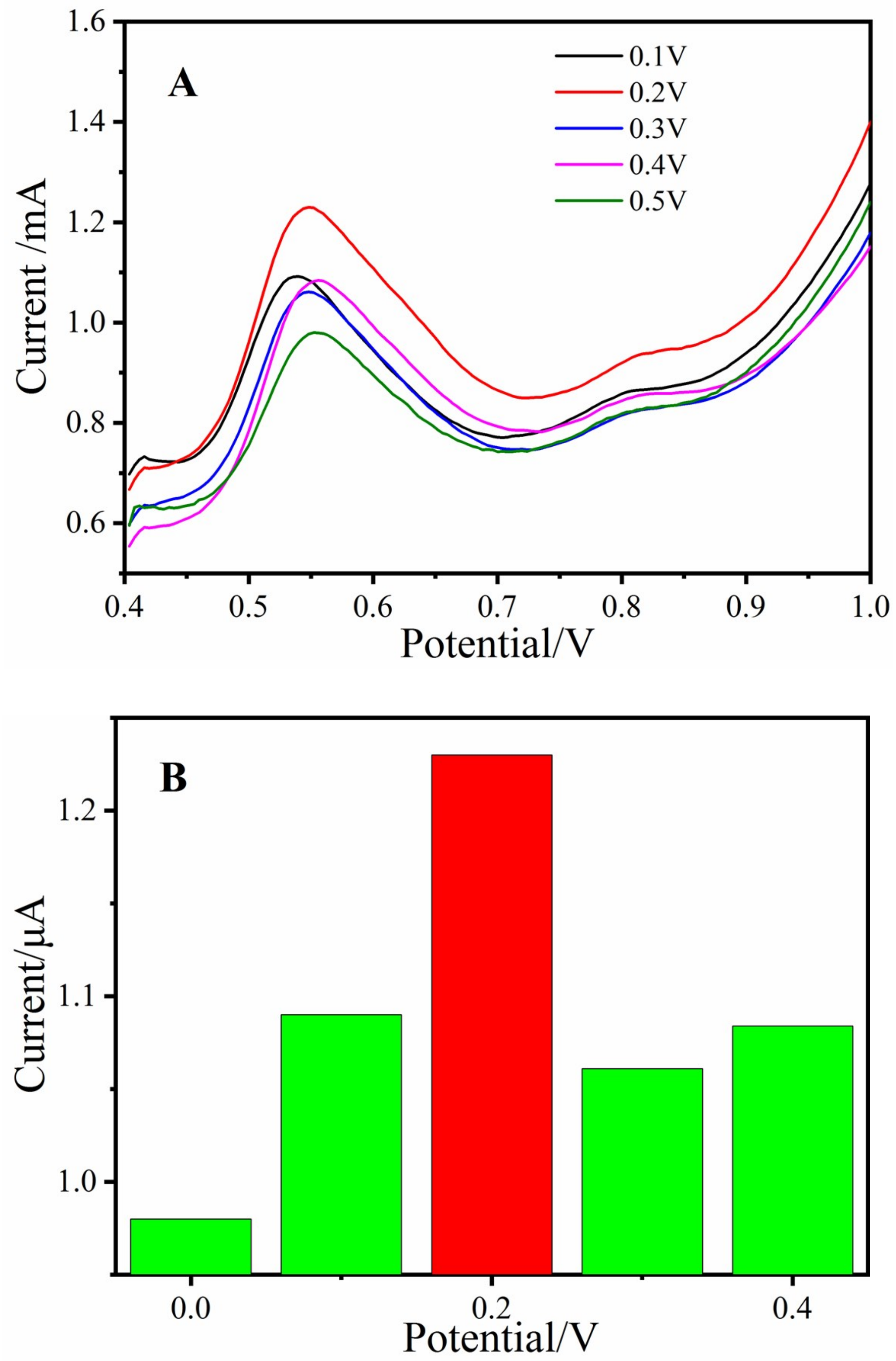

Fig. S7 (A) DPV curves and (B) peak currents of 1×10^{-6} M RAC in PBS with accumulation potentials in the range of 0 to 0.4 V.

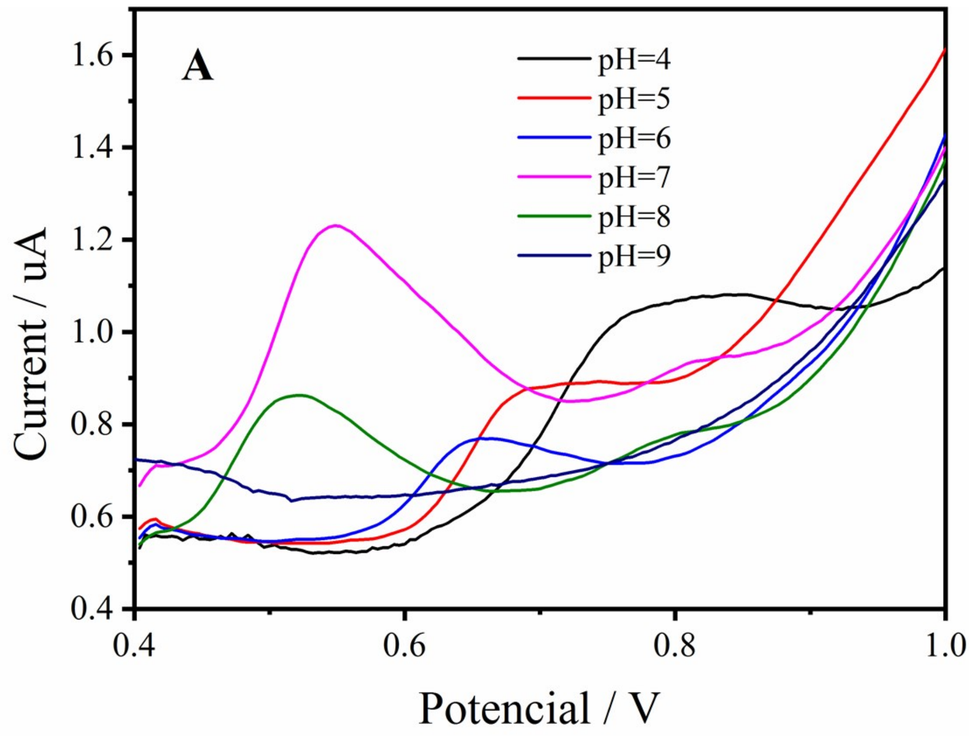

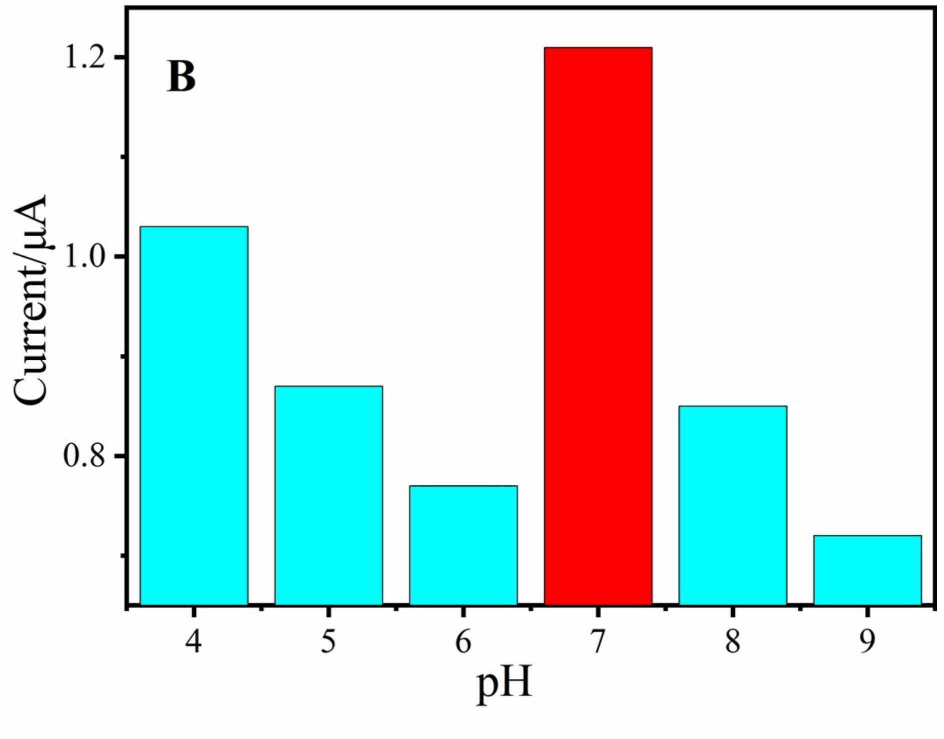

Fig. S8 Effect of solution pH on (A) DPV and (B) electrochemical response of RAC (1×10^{-6} M) on Ag_2TPPS_4/AgNPs/ng-C_3N_4/GCE.

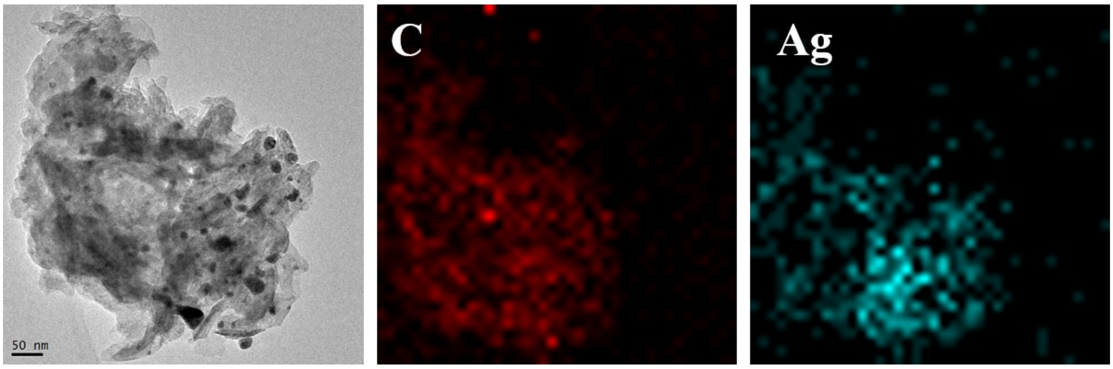

Fig. S9 Elemental mapping of Ag_2TPPS_4/AgNPs/ng-C_3N_4/GCE.

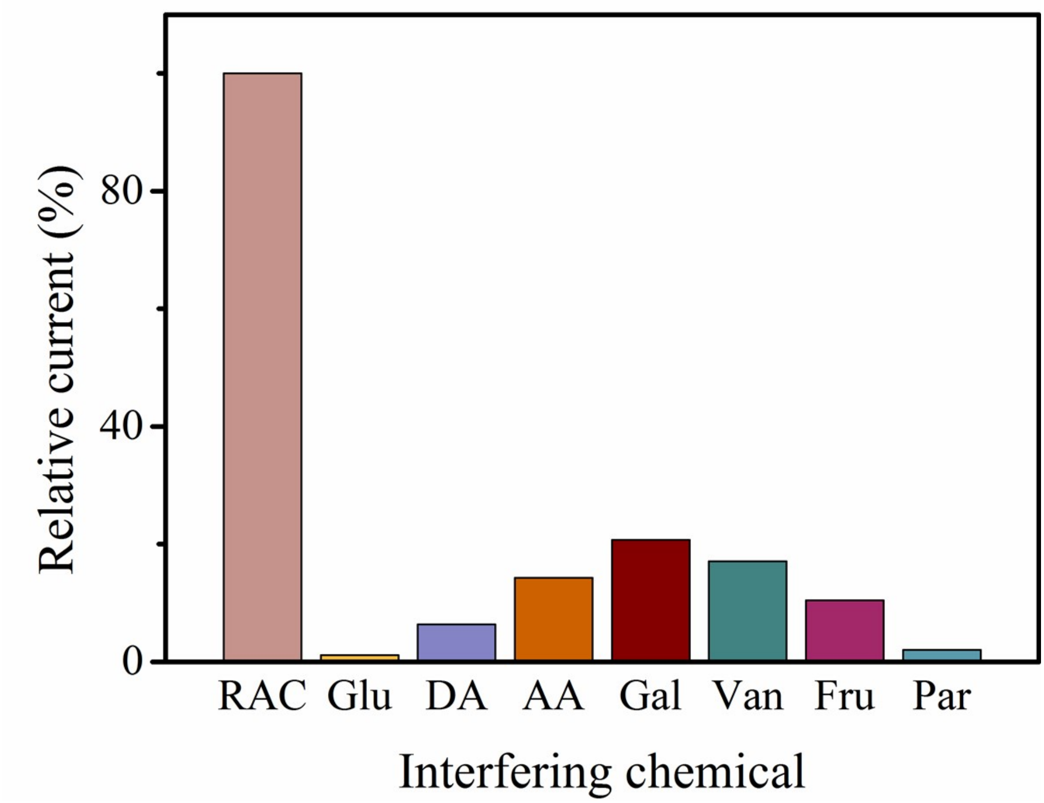

Fig. S10 Current response of Ag_2TPPS_4/AgNPs/ng-C_3N_4/GCE in 50 μM RAC with 500 μM interferences.

Table S1 Zeta potentials of Ag_2TPPS_4/AgNPs/ng-C_3N_4, AgNPs/ng-C_3N_4, RAC, AgNPs/ng-C_3N_4+RAC and Ag_2TPPS_4/AgNPs/ng-C_3N_4+RAC in H_2O.

Samples	Zeta potential (mV)
AgNPs/ng-C_3N_4	-2.13
Ag_2TPPS_4/AgNPs/ng-C_3N_4	-42.2
RAC	1.26
AgNPs/ng-C_3N_4+RAC	7.69
Ag_2TPPS_4/AgNPs/ng-C_3N_4+RAC	-15.6

Table S2 Comparison of analytical parameters for RAC oxidation at $Ag_2TPPS_4/AgNPs/ng-C_3N_4$ electrode with reported works.

Methods	Linear range (mol/L)	Limit of detection (mol/L)	Ref.
$Bi_2Te_3@g-C_3N_4$ BNs	1.5×10^{-8} – 4.56×10^{-4}	1.77×10^{-9}	[1]
Fe_3O_4-RGO	1.0×10^{-5} - 1.0×10^{-4}	1.3×10^{-8}	[2]
NPVMo/ZrO_2	3.0×10^{-6} - 5×10^{-5}	9.3×10^{-7}	[3]
quartz crystal microbalance	2.5×10^{-6} - 1.5×10^{-4}	1.17×10^{-6}	[4]
OMC	8.5×10^{-8} - 8×10^{-6}	6×10^{-8}	[5]
$Ag_2TPPS_4/AgNPs/ng-C_3N_4$	1×10^{-7} - 1.2×10^{-5}	5.1×10^{-8}	This work

Table S3 Amount and recovery rate of RAC in milk samples.

Samples	Added (nM)	Found (nM)	Recover (%)
1	5000	4952	99%
		4762	95%
		4619	92%
2	1000	1043	104%
		1062	106%
		990	99%
3	100	114	114%
		110	110%
		105	105%

References

1 U. Rajaji, T. W. Chen, S. Chinnapaiyan, S.-M. Chen, M. Govindasamy, *Anal. Chim. Acta.*, 2020, **1125,** 220-230.

2 Y. Poo-arporn, S. Pakapongpan, N. Chanlek, R.P. Poo-arporn, *Sens. Actuators B Chem.*, 2019, **284**, 164-171.

3 L. Zhang, Q. Wang, Y. Qi, L. Li, S. Wang, X. Wang, *Sens. Actuators B Chem.*, 2019, **288**, 347-355.

4 L. J. Kong, M. F. Pan, G. Z. Fang, X. l. He, Y.-k. Yang, J. Dai, S. Wang, *Biosens. Bioelectron.*, 2014, **51**, 286-292.

5 X. Yang, B. Feng, P. Yang, Y. Ding, Y. Chen, J. Fei, *Food Chem.*, 2014, **145**, 619-624.
